# 5,6-Didehydroginsenosides from the Roots of *Panax notoginseng*

**DOI:** 10.3390/molecules15118169

**Published:** 2010-11-11

**Authors:** Jian-Bo Wan, Qing-Wen Zhang, Si-Jia Hong, Jia Guan, Wen-Cai Ye, Shao-Ping Li, Ming-Yuen Simon Lee, Yi-Tao Wang

**Affiliations:** 1 Institute of Chinese Medical Sciences, University of Macau, Taipa, Macao, China; E-Mails: wjbcpu@hotmail.com (J.-B.W.); brendajia0901@hotmail.com (S.-J.H.); ya67402@umac.mo (J.G.); spli@umac.mo (S.-P.L.); simonlee@umac.mo (M.-Y.S.L.); 2 Department of Medicine, Massachusetts General Hospital and Harvard Medical School, Boston, MA 02114, USA; 3 Institute of Traditional Chinese Medicine and Natural Products, College of Pharmacy, Jinan University, Guangzhou, 510632, China; E-Mail: chywc@yahoo.com.cn (W.-C.Y.)

**Keywords:** *Panax notoginseng*, triterpene saponins, didehydroginsenosides

## Abstract

Two minor novel dammarane-type saponins—5,6-didehydroginsenoside Rd (**1**) and 5,6-didehydroginsenoside Rb1 (**2**)—were isolated from the dried roots of *Panax notoginseng* along with sixteen known saponins. The structures of the new compounds were elucidated on the basis of spectroscopic and chemical methods.

## 1. Introduction

Root of *Panax notoginseng* (Burk.) F. H. Chen (Araliaceae), also called Sanqi, is a well-known herb commonly used as a medicine and dietary supplement. It has been cultivated commercially in the southwest regions of China, especially in the Wenshan region, Yunnan Province. *P. notoginseng* has been used in China as a drug for the treatment of haemoptysis, haemostatic and haematoma for more than 400 years [1]. Current pharmacological studies revealed that *P. notoginseng* and its ingredients possess anticarcinogenic [2,3], immunoregulatory [4], anti-inflammatory [5], anti-arrhythmic [6], hepatoprotective [7] properties, as well as protective effects on cardiovascular and cerebrovascular systems [1,8,9]. Drammarane type saponins are considered as the major bioactive constitutes in *P. notoginseng* [1,10,11,12]*.*

**Figure 1:**
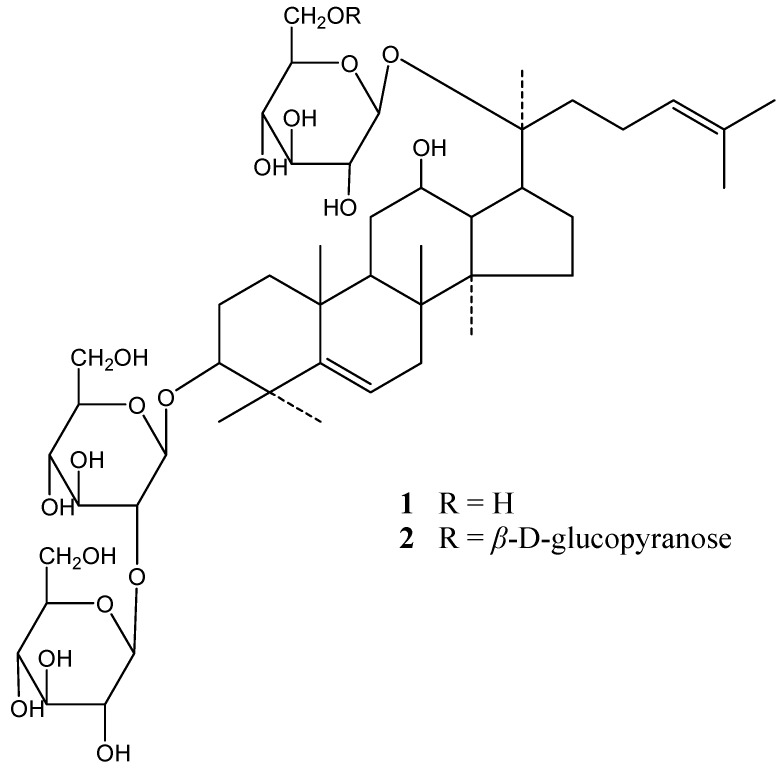
Structures of compounds **1** and **2**.

During our studies on the screening of bioactive ingredients from medicinal herbs with activity against vascular inflammation, the glycosidic fraction from the roots of *P. notoginseng *was observed to reduce atherosclerotic lesions in apoE deficient mice, an effect which may be responsible for its inhibitory action on vascular inflammation [13]. During further study on this fraction, two novel triterpene saponins, 5,6-didehydroginsenoside Rd (**1**) and 5,6-didehydroginsenoside Rb1 (**2**) (Figure 1), were isolated from the methanolic extract of the roots together with sixteen known dammarane-type saponins. We report herein the structure elucidation of these components.

## 2. Results and Discussion

The methanol extract of the dried roots of *P. notoginseng* was subjected to D-101 macroporous adsorption resin separation and chromatographed repeatedly on ordinary and reverse phase silica-gel columns, and finally on preparative high-performance liquid chromatography (prep-HPLC) to afford eighteen saponins **1-18**. The spectroscopic data of compounds **3**-**18** were identical with those of notoginsenosides R1 (**3**), R2 (**6**) [14], Fa (**10**) [15], R4 (**11**) [16], T (**12**) [17], K (**17**) [18], ginsenosides Rg1 (**4**) [19], Re (**5**), Rg2 (**7**) [14,20], Rh1 (**8**) [14], Ra3 (**13**) [21], Rb1 (**14**), Rd (**16**) [22], chikusetsusaponin L5 (**9**) [23], malonyl-ginsenoside Rb1 (1**5**) [24] and quinquenoside R1 (**18**) [25], which were isolated previously from *Panax* genus plants. Compounds **9, 15 **and **18 **were thus isolated for the first time from the roots of *P. notoginseng*. The structures of the new saponins **1-2** were determined on the basis of spectroscopic and chemical methods described as follows. 

Compound **1** was obtained as a white amorphous solid. The negative ion ESI-MS of **1 **showed a quasimolecular ion [M-H]^-^ at *m/z *943, consistent with a molecular formula of C_48_H_80_O_18_. Compound **1** exhibited a mass difference of 2 in comparison to ginsenoside Rd (**16**). Acid hydrolysis of **1** afforded glucose as the only sugar component. The ^1^H-NMR spectrum of **1 **displayed signals due to eight tertiary methyls at *δ*_H_ 0.91, 0.99, 1.09, 1.42, 1.49, 1.58 (2 × CH3) and 1.61, and two olefinic protons at *δ*_H_ 5.59 and 5.24 for the aglycon moiety. The ^1^H- and ^13^C-NMR (Table 1) spectra of **1** showed signals assignable to three *β*-D-glucopyranosyl moieties [*δ*_H_ 4.87 (1H, d, *J* = 7.5 Hz, H-1'), 5.17 (1H, d, *J* = 7.8 Hz, H-1''') and 5.34 (1H, d, *J* = 7.1 Hz, H-1'')]. Further analysis of the NMR data suggested that the aglycon of **1** could be a substituted dammarane-type triterpene. The ^1^H- and ^13^C-NMR resonances of the sapogenin moiety of **1** are very similar to that of the protopanaxadiol bisdemosidic saponins such as ginsenoside Rd and Rb1 [16,26,27], except for signals of ring B, C-4, C-11, C-19, C-28 and C-29 and the presence of an additional olefin group (*δ*_H_ 5.59 and *δ*_C_ 119.8 and 147.4), suggesting that **1** could be a dehydroprotopanaxadiol bisdemosidic saponin. The additional double bond was assigned to C-5 and C-6 from the correlations in HMBC spectra between C-5 (*δ*_C_ 147.4) and 19-H_3_ (*δ*_H_ 1.09), 28-H_3_ (*δ*_H_ 1.49) and 29-H_3_ (*δ*_H_ 1.42), and between H-6 (*δ*_H_ 5.59) and C-5 (*δ*_C_ 147.4), C-4 (*δ*_C_ 43.0) and C-10(*δ*_C_ 37.3). The ^13^C-NMR data also suggested **1** had the same sugar chains and glycosidic locations with that of ginsenoside Rd (27). Location of a *β*-sophorosyl moiety at C-3 position of the aglycon was confirmed by HMBC correlations between carbon signals at *δ*_C_ 88.0 (C-3), 104.9(C-1'), 83.7(C-2') and proton signals at *δ*_H_ 4.87 (H-1'), 3.30 (H-3), 5.34 (H-1''), respectively. Similarly, a long-range correlation between the carbon signal at *δ*_C_ 83.3 (C-20) and the proton signal at *δ*_H_ 5.17 (H-1''') ascertained that a *β*-D-glucopyranosyl unit is located at the C-20 of the sapogenin. Thus, the structure of compound **1** was determined as 3-*O*-*β*-D-glucopyranosyl-(2-1)-*β*-D-glucopyranosyl 5,6-didehydro-20(*S*)-protopanaxadiol 20-*O*-*β*-D-glycopyranoside (5,6-didehydroginsenoside Rd ).

Compound **2** was obtained as white amorphous powder. The spectra of **2** were in part similar to those of 1. The negative ion ESI-MS of **2** displayed quasimolecular ion peak at m/z 1,105 [M-H]^-^, consistent with a molecular of C_54_H_90_O_23_, which suggested the presence of an extra hexose unit in **2 **compared to **1**. Acid hydrolysis of **2** afforded D-glucose. Further comparison of the ^13^C-NMR data of **2 **(Table 1) with that of **1** and ginsenoside Rb1 revealed that the sapogenin and sugar chains of **2** were the same as that of **1** and ginsenoside Rb1 [16], respectively. The above finding was supported by the HMBC experiment. Long range correlation signals were observed between carbon resonances at C-5 (*δ*_C_ 147.4) and 19-H_3_ (*δ*_H_ 1.09), 28-H_3_ (*δ*_H_ 1.49) and 29-H_3_ (*δ*_H_ 1.42), between H-6 (δ_H_ 5.59) and C-5 (*δ*_C_ 147.4), C-4(*δ*_C_ 43.0) and C-10(*δ*_C_ 37.3), between H-1''(*δ*_H_ 5.34) of terminal glucose and C-2' (*δ*_C_ 83.6) of inner glucose, and between H-1' (*δ*_H_ 4.88) of inner glucose and C-3 (*δ*_C_ 87.9) of the aglycon. Moreover, HMBC spectrum revealed correlation between H-1'''' (*δ*_H_ 5.04) of additional terminal glucose and C-6''' (*δ*_C_ 70.3) of the bridging glucose as well as the correlation between H-1''' (*δ*_H_ 5.12) of the bridging glucose and C-20 (*δ*_C_ 83.4) of the aglycon. All above evidence led the identification of **2** as 3-*O*-*β*-D-glucopyranosyl-(2-1)-*β*-D-glucopyranosyl-5,6-didehydro-20(*S*)-protopanaxadiol-20-*O*-*β*-D-glucopyranosyl-(6-1)-β-D-glucopyranoside (5,6-didehydroginsenoside Rb1).

**Table 1:** ^13^C NMR data of compounds **1** and **2** (in C_5_D_5_N, *δ *in ppm).

C	1	2	C	1	2
1	39.7	39.7	3- *O*-Sugar	glc	glc
2	27.0	27.0	1'	104.9	104.9
3	88.0	87.9	2'	83.7	83.6
4	43.0	43.0	3'	78.0	78.0
5	147.4	147.4	4'	71.7	71.7
6	119.8	119.7	5'	78.3	78.3
7	34.8	34.8	6'	63.0	62.9
8	37.2	37.2		glc(1-2)	glc(1-2)
9	47.2	47.1	1"	106.2	106.1
10	37.3	37.3	2"	77.1	77.0
11	32.6	32.4	3"	78.0	78.2
12	70.0	69.9	4"	71.7	71.8
13	49.5	49.5	5"	78.3	78.3
14	51.0	51.0	6"	62.9	62.8
15	31.3	31.2	20- *O*-sugar	glc	glc
16	26.7	26.7	1'"	98.3	98.1
17	51.9	51.8	2'"	75.2	75.2
18	18.0	18.0	3'"	79.2	79.1
19	20.4	20.4	4'"	71.7	71.7
20	83.3	83.4	5'"	78.3	77.0
21	22.5	22.5	6'"	62.8	70.3
22	36.4	36.4			glc(1-6)
23	23.3	23.2	1''"		105.3
24	126.0	126.0	2''"		74.9
25	130.9	131.1	3''"		78.3
26	25.7	25.7	4''"		71.8
27	17.8	17.9	5''"		78.3
28	28.1	28.1	6''"		62.8
29	24.2	24.1			
30	16.9	16.9			

## 3. Experimental

### 3.1. General

ESI mass spectra were recorded on an LC-MSD trap VL mass spectrometer (Agilent Technologies, Palo Alto, CA, USA). NMR spectra were recorded on a Bruker AV-400 spectrometer (C_5_D_5_N used as solvent and TMS as an internal standard). Column chromatography was performed with D-101 macroporous absorption resin (Haiguang Chemical Industrial Company, Tianjin, China) and silica gel (200–300 mesh, Qingdao Marine Chemical Group Co., Qingdao, China). Medium Pressure Liquid Chromatography (MPLC, Büchi, Switzerland) and Agilent 1100 Series prep-HPLC apparatus (Palo Alto, CA, USA) were also used for further isolation. For detection, HPLC was performed on an Agilent 1100 series HPLC apparatus. A Zorbax SB-C18 column (250 mm × 4.6 mm, I.D., 5 μm) and a Zorbax SB-C18 guard column (12.5 mm × 4.6 mm I.D., 5 µm) were used at a 25 °C. The mobile phase consisted of water (A) and acetonitrile (B), the detection wavelength was set at 203 nm. D-Glucose and pyridine (Reagent Plus, ≥99%) were purchased from Sigma (St. Louis, MO, USA). Trifluoroacetic acid (TFA, 99%) was purchased from Riedel-de Haën (Seelze, Germany). GC-MS was performed on an Agilent 6890 gas chromatography instrument coupled with an Agilent 5973 mass spectrometer (Agilent Technologies, Palo Alto, CA, USA). 

### 3.2. Plant Material

The roots of *P. notoginseng *were collected in Wenshan region, Yunnan province, China. The botanical origin of material was identified by Dr. Xiu-ming Cui, Wenshan Prefecture Sanqi Research Institute, Yunnan Province. The voucher specimen was deposited at the Institute of Chinese Medical Sciences, University of Macau, Macao, China.

### 3.3. Extraction and Isolation

Air-dried, powdered roots (2.5 kg) of *P. notoginseng* were extracted three times with boiling methanol (20 L × 3; 4 h, 2 h and 2 h, respectively). After filtration, excess solvent was removed on a rotary evaporator (Büchi Labortechnik AG, Switzerland) at 60 ºC. The residue was suspended in distilled H_2_O, and subjected to the D-101 macroporous absorption resin eluting with H_2_O, 20% EtOH and 80% EtOH (v/v, aqueous-EtOH) in sequence. The 80% EtOH fraction was collected as total crude saponin. The total crude saponin (210 g) was reloaded on the macroporous column and eluted with H_2_O, 35% EtOH and 80% EtOH (v/v, aqueous-EtOH) [28] to provide a protopanaxatriol saponin fraction (PTS, I), a protopanaxadiol saponin fraction (PDS, II) and a mixture of PTS and PDS (III). The three fractions were dried in the rotary evaporator and weighed 112 g, 94 g and 14 g, respectively. Fraction **I** (14.5 g) was separated on silica gel column eluting with EtOAc-MeOH-H_2_O (90:10:1, 85:15:1, 80:20:2 and 75:25:2) and followed MPLC to give the pure compounds notoginsenoside R1 (**3, **1.35 g), ginsenoside Rg1 (**4**, 7g,) and ginsenoside **5** (Re, 0.52 g). Fraction II (30 g) was loaded on a silica gel column, and eluted with EtOAc-MeOH-H_2_O (85:15:3, 80:20:3, 70:30:3) to afford ginsenoside Rb1 (**14**, 5.7 g) and two major fractions (IV and V). Fraction IV was separated by Prep-HPLC on a C_18_ column with MeOH-H_2_O (68:32) to give ginsenosides R2 (**6**, 6 mg), Rg2 (**7**, 10 mg) and Rh1 (**8**, 3 mg). Fraction VI was separated by Prep-HPLC with MeOH-H_2_O (76:24) on the same C_18_ column as above and resulted in the isolation of chikusetsusaponin L5 (**9**, 10 mg), malonyl-ginsenoside Rb1 (**15**, 5 mg), ginsenoside Rd (**16**, 1.5 g), notoginsenoside K (**17**, 40 mg), quinquenoside R1 (**18**, <1 mg), and 5,6-didehydro-ginsenoside Rd (**1**, 2 mg). Fraction III was separated by Prep-HPLC with MeOH-H_2_O (68:32) on the same C_18_ column as above and resulted in the isolation of notoginsinoside T (**12**, 42 mg), R4 (**11**, 68 mg), Ra3 (**13**, 40 mg), Fa (**10**, 95 mg) and 5,6-didehydroginsenoside Rb1 (**2**, 26 mg). Two mg of quinquenoside R1 (**18**) and 20 mg of 5,6-didehydroginsenoside Rd (**1**) were isolated from the remaining fraction II by the same procedure described above. 

*5,6-Didehyroginsenoside Rd* (**1**) was obtained as a white amorphous solid. [*α*]^25^_D_ = +3.8° (*c* 0.08, MeOH). ESI-MS *m/z* 979.5 [M+Cl]^ −^, 943 [M−H]^−^, 781 [M-H-Glc]^−^, 619 [M-H-2Glc]^−^, 457 [M-H-3Glc]^−^. ^1^H-NMR (C_5_D_5_N, 400 MHz) *δ*_H_: 0.91 (3H, s, H_3_-18), 0.99 (3H, s, H_3_-30), 1.09 (3H, s, H_3_-19), 1.42 (3H, s, H_3_-29), 1.49 (3H, s, H_3_-28), 1.58 (6H, s, H_3_-26,27), 1.61 (3H, s, H_3_-21), 3.30 (1H, dd, *J* = 11.6, 4.4 Hz, H-3), 5.24 (1H, t-like, H-24), 5.59 (1H, m, H-6), 4.87 (1H, d, *J* = 7.5 Hz, H-1' of glc), 5.17 (1H, d, *J* = 7.8 Hz, H- 1''' of glc), 5.34 (1H, d, *J* = 7.8 Hz, H-1'' of glc). ^13^C-NMR: see Table 1.

*5,6-Didehyroginsenoside Rb1* (**2**) was obtained as a white amorphous solid. [*α*]^25^_D_ = +16.9° (*c* 0.05, MeOH). ESI-MS *m/z* 1141.6 [M+Cl]^−^; 1105 [M−H]^−^, 943 [M−H−Glc]^−^, 925 [M−H−Glc−H_2_O]^−^, 781 [M−H−2Glc]^−^, 763 [M−H−2Glc−H_2_O]^−^, 619 [M−H−3Glc]^−^. ^1^H-NMR (C_5_D_5_N, 400 MHz) *δ*_H_: 0.92 (3H, s, H_3_-18), 0.99 (3H, s, H_3_-30), 1.09 (3H, s, H_3_-19), 1.42 (3H, s, H_3_-29), 1.49 (3H, s, H_3_-28), 1.58 (3H, s, H_3_-26), 1.64 (3H, s, H_3_-21), 1.64 (3H, s, H_3_-27), 3.31 (1H, dd, *J* = 11.5, 4.2 Hz, H-3), 5.31 (1H, t-like, H-24), 5.59 (1H, br s, H-6), 4.88 (1H, d, *J* = 7.3 Hz, H-1' of glc), 5.04 (1H, d, *J* = 7.7 Hz, H-1'''' of glc), 5.12 (1H, d, *J* = 7.8 Hz, H- 1''' of glc), 5.36 (1H, d, *J* = 7.8 Hz, H-1'' of glc). ^13^C-NMR: see Table 1.

### 3.4. Acidic Hydrolysis of Compounds *1* and *2*

Each sample (1 mg) was hydrolyzed with 2 mol·L^-1^ TFA (1 mL) at 100 ºC for 2 h in a sealed glass tube with a screw cap which was filled with pure nitrogen gas. The hydrolyzed solution was evaporated to dryness under 45 ºC and then methanol (1 mL) was added for further evaporation and complete removal of TFA. The hydrolysate was treated with 1 mL hydroxylamine hydrochloride-pyridine solution (20 mg·mL^-1^) at 90 ºC for 30 min in a sealed glass tube fitted with a screw cap. After cooling to room temperature, acetic anhydride (1 mL) was added and heating continued for another 30 min in the resealed tube. The cooled solution was evaporated to dryness under reduced pressure at 45 ºC. The residue was dissolved in dry chloroform (2 mL). The solution was filtered through a 0.45 μm syringe filter (Agilent Technologies) prior to injection into GC-MS system. GC-MS was carried out on a HP-5MS capillary column (30 m × 0.25 mm, i.d.) coated with 0.25 μm film 5% phenyl methyl siloxane. The column temperature was set at 175 ºC and held for 7 min, then programmed at 5 ºC·min^-1^ to 185 ºC and held for 5 min, then at 4 ºC·min^-1^ to 230 ºC. Split injection (2 μL) with a split ratio of 1:50 was applied. High purity helium was used as carrier gas with flow rate of 1.0 mL·min^-1^. The mass spectrometer was operated in electron-impact (EI) mode, the scan range was 40–550 amu, the ionization energy was 70 eV and the scan rate was 2.89 s per scan. The inlet, ionization source temperature were 250 and 280 ºC, respectively. The same reaction and analysis were applied for standard sugar (D-glucose). The D-glucose derivative showed a peak at *t*_R_ 12.5 min. As a result, D-glucose was detected from both **1** and **2**.

## 4. Conclusions

In this study, the glycosidic fraction from the roots of *P. notoginseng *was investigated. Two novel saponins, 5,6-didehydroginsenoside Rd (1) and 5,6-didehydroginsenoside Rb1 (2), were isolated from the roots of *P. notoginseng*, along with sixteen known saponins. This result will be helpful to better understand the chemical components of *P. notoginseng.*
